# Food Hygiene Practices at the Ghana School Feeding Programme in Wa and Cape Coast Cities

**DOI:** 10.1155/2020/9083716

**Published:** 2020-05-12

**Authors:** Kate Bigson, Edward Ken Essuman, Comfort Worna Lotse

**Affiliations:** ^1^Department of Hotel, Catering and Institutional Management, Wa Polytechnic, Wa, Ghana; ^2^Department of Nutrition and Dietetics, University of Health and Allied Sciences, Ho, Ghana; ^3^Department of Public Health Nursing, University of Health and Allied Sciences, Ho, Ghana

## Abstract

**Objective:**

The integrity and the wholesomeness of the food served to school pupils cannot be overlooked, especially when one considers the magnitude of health and sanitation issues that are plaguing the West African nations. This study aimed to investigate some of the personal hygiene practices by the pupils and the hygienic conditions in which food is cooked and served to these school-going children under the Ghana School Feeding Programme (GSFP).

**Design:**

A cross-sectional and descriptive survey research designs were used in the study. Purposive and simple random sampling techniques were employed in selecting participants. *Participants*. There were 720 respondents for the study, comprising 600 pupils, 60 teachers, and 60 kitchen staff members from 20 schools. Information was obtained using questionnaire, observation, and unstructured interview instruments.

**Results:**

Findings from the study revealed that the majority of pupils (92% in Wa and 65% in Cape Coast) did not wash their hands with soap under running water. No hand washing centers for pupils were also seen in most of the schools studied. Majority of the cooks did not have health certificate, and neither had attended any in-service training in two years. In both Wa and Cape Coast municipal schools, none of the kitchen staff admitted that pupils and teachers ever complained about the meals they served to the pupils.

**Conclusion:**

The GSFP in basic schools forms part of the integral diet of the school children; hence, provision of good quality food can affect the health, learning, and physical activities of these children. Observational checklist revealed that most of the kitchen staff do not strictly adhere to basic food hygiene practices, and this affects the wholesomeness of the food served to the children. There is, therefore, a need for kitchen staff training on hygiene and food preparation practices.

## 1. Introduction

When the Ghana School Feeding Programme (GSFP) started in the year 2005, it gave the impression to be a quick-win intervention against hunger and school drop-out. This programme offers one hot nutritious meal each day for school children in some selected public schools funded by the government. However, ten years down the lane, the programme seems to be fraught with difficulties in its implementation. Unanswered issues cited by critics of the programme include the nutritional quality of meals served, the conditions under which food ingredients of the GSFP are procured and stored [1, 2], and the hygienic conditions under which meals are cooked, served, and eaten (unpublished results).

The health of the children, the safety of the food ingredients used in the meal preparation [3], and the hygienic conditions the products run through before they get onto the plates of these children are of the essence because hygiene is a major challenge in Ghana. Currently, there is no system designed to check the quality and safety of meals fed to the children enrolled under the GSFP. Sulemana et al. [4] have also reported on school kitchens being in the open air under trees or in temporary structures for protection against the rains. In both situations, food has to be cooked either in a classroom or on the veranda (structure attached to the exterior of the school building).

This is counterproductive to the achievement of the goals of GSFP, as it disrupts teaching and also affects the maintenance of a hygienic environment for food preparation. Suleman et al. [4] further revealed that 60% of the schools visited did not have adequate stock of plates, cups, and spoons. This implies that some pupils have to wait and reuse plates and spoons used by their friends after washing them. As a result, pupils eat in turns creating prolonged lunch breaks and reduced time spent in class by the pupils. It also subjects the children to possible cross-contamination of diseases and other contaminants.

According to Bolton [5], young children are more at risk of contracting a foodborne illness because they have not yet built adequate immune system (the body's defence system against illness) to deal with some diseases. Hence, practicing personal hygiene is of utmost important since contamination of food mostly comes from contact with faecal material or microorganisms delivered by contact with contaminated hands, cooking surfaces and utensils, the soil on the ground, or improperly cleaned dishes and cutlery. Research shows that, if widely practiced, hand washing with soap could reduce diarrhoea by almost 50% [6] and respiratory infections by nearly 25% [7]. Veronica Bucket (named after Veronica Bekoe, a Ghanaian biological scientist, and comprised of a bucket and a basin on top of a wooden stand) is being used in some schools to act as ministation for hand washing and bowl washing.

Hoddinott and Yohannes [8] take the view that a person handling food should be clean and clothed in fresh apparel. FAO [9] stated that there are so many ways to put others at risk when handling food. Recent statistics in some states have confirmed that over half of foodborne illnesses can be traced directly back to food handlers and improper hygiene [6]. Following a few personal hygiene rules can help minimize food safety problems. According to Victora et al. [10], failure on the part of food servers to wash their hands (or to wash them properly) is one of the biggest threats to consumers. There is so much contamination that can occur in a kitchen, such as improper handling of raw foods and cooked foods without sanitizing the hands or utensils.

Information regarding the health and safety of the food served under the GSFP is scarce. Data on hygienic conditions of the kitchens and water quality for the food preparation is scanty. However, the integrity and the wholesomeness of the food served to the school pupils cannot be overlooked, especially when one considers the magnitude of health and sanitation issues that are plaguing the West African nations. Therefore, the specific objectives of this study were to (1) identify bowl and hand washing practices by the pupils and sources of water for cooking meals under the GSFP and (2) assess the hygienic conditions under which food is cooked and served to the pupils. This study was part of a project to investigate the nutritional quality and safety of meals served under the GSFP in Wa and Cape Coast municipal schools in Ghana.

## 2. Materials and Methods

### 2.1. Study Area

The study was conducted in two major capital towns, Wa and Cape Coast in the Upper West and Central Region of Ghana, respectively. The choice of schools for the study was based on Ghana population and housing census 2010 [11] data that points to the three northern regions (Upper East, Upper West, and Northern Regions) and the Central Region as the poorest regions in Ghana. This, therefore, led to the selection of Wa and Cape Coast in the research study. Schools in Cape Coast include 9 public senior high institutions, 3 private senior high institutions, and 120 junior high schools, primary schools, and preschools belonging to both public and private sectors. There are also 14 schools under the school feeding programme. Each school had an average of 300 students. Also, the Wa Metropolis has 68 schools participating in the Ghana School Feeding Programme (GSFP) with an average population of 300 pupils in a school.

### 2.2. Study Population

The target population included all teachers, pupils (upper primary), and kitchen staff found within schools benefitting from GSFP in the Wa Municipality and Cape Coast Metropolis. The choice of teachers, pupils, and kitchen staff, therefore, enabled the generation of first-hand information on the conditions under which food are cooked, served, and eaten under the GSFP.

### 2.3. Sample Size

A total of 720 respondents participated in the study. This sample size was computed using Graph Pad Prism Version 16, statistical software. The following parameters were used: standard normal variance (at 5% type 1 error; *P* < 0.05), a critical *z*-score of 1.96% (at 1% type 1 error; *P* < 0.01), with 95% ± 2.58 confidence interval.

### 2.4. Study Design, Sampling Techniques, Instrumentation, and Analysis

A cross-sectional and descriptive survey research designs were used to identify the existence and extent of disparity that might be in the GSFP in the Wa and Cape Coast schools. Nonprobability sampling using purposive sampling was employed to identify public schools benefitting from the GSFP in both Wa and Cape Coast. Probability sampling using simple random sampling method was used to select 12 schools out of the 68 schools under the school feeding programme from Wa, while 8 out of 14 schools were selected from the Cape Coast schools. In these schools, 15 pupils each were selected from classes 5 and 6 making a total of 30 pupils per school for the survey. Where the class had a register, the third consecutive name was selected. Where the class register was not immediately available, the pupils were randomly sampled (up to 15 per class). The upper primary pupils were chosen for this study because they could read and write and will better understand the questions being asked without much assistance. For the teachers and headmasters or headmistresses, each headmaster or headmistress was automatically selected from each school including a teacher specially assigned to see to the GSFP. One teacher was randomly selected from either class 5 or class 6 from each school. The total heads of schools and teachers were 60. One head, matron, or caterer in charge of cooking food for these school children was automatically selected and 3 cooks were randomly selected from each schools' kitchen staff making a total of 60 as shown in Table 1.

A pretested standardized validated questionnaire, consisting of closed-ended questions with multiple-choice answers and open-ended questions, was used to solicit information on the hygienic conditions of meals prepared and served to the pupils. The reliability test carried out during the pretest produced Cronbach Alpha values of ≥0.75 for all the sections. According to Pallant [12], Cronbach Alpha values of more than 0.7 imply that the instrument is reliable.

An unstructured observational guide was developed to assist in identifying hygienic practices of pupils, teachers, and kitchen staff. Data were analyzed using Statistical Package for Social Sciences software version 22.0 [13]. Descriptive statistics were used to summarize the data into frequency and percentage.

## 3. Results and Discussion

### 3.1. Demographic Characteristics of Respondents

Of the 360 pupils studied from Wa schools, the minimum, mode, mean, and maximum age were 10, 13, 14, and 20 years, respectively; for Cape Coast schools, they were 9, 12, 12, and 16 years, respectively, as shown in Table 2. Also, Wa schools recorded 234 males and 126 females while Cape Coast schools had 134 males and 106 females. Health certificate verification was also done to ascertain if the kitchen staff were healthy to qualify them to cook for the pupils. Results showed that they were mostly healthy. However, a quite sizeable number of the kitchen staff did not have the health certificates. More than half of the number of caterers in Wa Municipality had nonformal education, one-third had basic education, and the rest had secondary or vocational training. This was contrary to the observations made in Cape Coast Metropolis where all of the caterers had secondary or vocational education.

### 3.2. Status of Hygienic Practices, Water Source, and Sanitation

When respondents were asked who washed the bowls from which school children ate, in Wa, the responses indicated that 98.0% of the bowls were washed by the school pupils and the statement was supported by 93.0% of their teachers, while 66.7% of the kitchen staff claimed they wash the bowls for the pupils (Table 3). In Cape Coast Metropolis school, responses indicated that 94.6% of the pupils washed their bowls and this was supported by 92.2% of the teachers. In contrast to these responses, 87.6% of the kitchen staff claimed they wash all the pupils' bowls. Due to inadequate water supply at these schools, pupils are made to wash the bowls and their hands in the same bowl of water. An additional issue that was observed was that some of the schools use outdoor latrines, and where there are no associated hand washing facilities, this could increase the risk of spread of faecal matter. A study conducted by Tetteh-Quarcoo et al. [14] identified *Aspergillus niger* from soapy water used by preschool children for hand washing. Hence, the use of communal bowl for washing bowls and hands could be a possible route for faecal contaminants to be present as much as *Aspergillus* which has been reported as a cause of invasive pulmonary aspergillosis [15].

To resolve the contradiction, field observation revealed that some of the pupils washed their bowls during break time but there were other schools where kitchen staff were also seen washing the bowls. One intriguing observation made was that, in most schools in Wa and Cape Coast, the feeding bowls were provided by the pupils except three schools in Wa where the school provided feeding bowls bought by contributions made by the pupils. Although, most of the pupils brought their bowls from the house, some were found washing their bowls after eating in the communal bowl meant for washing bowls and hands.

Table 4 shows that, in Wa, 92.22% of the pupils supported by 72.22% of the teachers and 88.89% of the kitchen staff claimed that the pupils wash their hands without soap under running water before eating. Also, in Cape Coast, 64.58% of the pupils supported by 72.22% of the teachers and 58.34% of the kitchen staff claimed that the pupils did not wash their hands with soap under running water before eating. Also, while some washed their hands under running water without soap, others washed their hands in basins of water with or without soap. However, some pupils correctly washed their hands under running water with soap while a significant number also did not wash their hands at all. Nevertheless, the level of awareness of hand washing among the pupils was good as almost all the pupils wash their hands before eating the meals served to them. However, with a definition of good hand washing practice being washing both hands with soap under running water, it was found that majority of the pupils in Wa and Cape Coast schools did not practice good hand washing techniques, a fault that need to be changed. Hand washing is a precautionary measure to protect against the spread of diseases and is one of the primary practices to reduce the transfer of bacteria from person to food contact surfaces. [16] It is an effective [6], feasible [17–19], and cost-effective [20] means of preventing gastroenteric infection worldwide.

Findings of the sources of water for hand washing and meal preparations revealed that the regular source was pipe-borne water, while others used local dug out wells. All of the respondents in Wa and Cape Coast (pupils, teachers, and kitchen staff) mentioned that they used pipe-borne water for cooking meals (Table 5). Other sources of water used included boreholes and local wells when the pipe-borne water was not available. Water is a critical raw material in food preparation and can create a public health risk when it is used for washing foods, incorporated in the food as an ingredient, and used for washing utensils and hands [21].

Most schools visited in Wa and Cape Coast had standing pipes on the school compounds from which pupils drank water. In one school in Wa, for example, parents had provided two poly-tanks which had been filled with water for pupils to drink and wash their hands. However, in another school in Wa, water had not been provided for the pupils to wash their hands or to drink and they had to buy from water vendors. They reported that the water they bought was not enough to drink, let alone to wash their hands. The insufficient water in some of the schools can compel some of the pupils not to wash their hands before eating their meal. This situation can also force the kitchen staff to reuse contaminated water for cleaning plates for eating by the pupils, and this can result in food contamination, thereby having a serious consequence on the children's health.

Findings, as represented in Table 6, indicate that majority of the pupils in both Wa and Cape Coast schools (30.8 and 40.8%, respectively) claimed that the hygienic conditions under which the meals were served to them were fair. The teachers in both schools also took the view that the meals were served under good hygienic condition. The kitchen staff, however, stated that the conditions were generally very good. Table 6 also revealed that the hygienic condition under which meals were eaten by the pupils in Wa was poor according to the majority (55.0%) of the pupils. The majority of the teachers however said it was good, and so did the kitchen staff. While in Cape Coast the majority of the pupils claimed that the conditions under which they eat their food were good, a quite significant percentage (32.5%) of the pupils thought otherwise. The teachers, as well as the kitchen staff, indicated that the conditions were quite good.

Food service providers are sources of foodborne illness outbreaks [22]. According to the World Health Organization (WHO) [23], food-handling personnel play an important role in ensuring food safety throughout the chain of food production and storage. The conditions under which the meals were prepared for the pupils were either good or fair according to the pupils, teachers, and kitchen staff (Table 6). Preparing food in unsanitary locations makes it susceptible to contamination by flies and domestic animals, and the link between other animals and diarrheal diseases has been reported [24].

It was observed that out of the 12 schools studied in Wa Municipality, 7 had their cooks wear uniforms and had their hairs also covered. The meals were dished into covered containers before sending them to the classrooms. In Cape Coast, the situation was somewhat different. Out of the 8 schools visited, only 2 had their cooks in uniforms. Most of the cooks did not cover their hair either, but when interviewed they said that they knew they should cover their hair. However, the meals were dished into covered containers before sending them to the classrooms. This is similar to a study by Monny et al. [25] in Ghana, where they found out that schools and other bodies provide education on food hygiene and safety for food vendors, which in this case are the kitchen staff or caterers who cook for the pupils in the school. Also, personal hygiene practices of the kitchen staff were observed to be generally good as the majority of them have acquired in-service training. The requirement of food handlers to wear aprons or to wear a hair covering during food preparation and serving is very important as this can help prevent the risk of cross-contaminating the food with germs [20].

The environment where the caterers cook the food was found not to be tidy. Dirty water made while cooking was also thrown in front of the cooking areas. Dirty cooking utensils were also seen piled up around the cooking areas in two of the schools, and litter was seen in the surrounding area. The presence of garbage at the place of cooking can attract more flies to the surroundings thereby increasing the chances of food being contaminated. These findings align with a study by Muide and Kuria [26] in Kenya who reported that those who cook and sell food mostly do not have garbage receptacle for waste collection, hence the disposal of garbage near their stalls. In one school, the cooks were seen sleeping in the cooking area with huge trash piled up in a corner of the kitchen as time ticks for the pupils to go for their lunch. In another school, the trash was spread around by toddlers of some of the kitchen staff while the meals were being served into bowls lined up on the floor.

In both Wa and Cape Coast municipal schools, animals like goats, dogs, and chicken were seen roaming around some of the open kitchens. This observation was encountered more often in Wa than in Cape Coast. There were also open gutters around some of the kitchens in Cape Coast with flies in them. Two schools had their kitchens built inside the school among the classrooms and the kitchens used gas to cook. Mice were seen during the observational tour in some of the kitchens in Cape Coast Metropolis schools, but this was denied by the kitchen staff. It is important for those cooking and preparing the food to do so away from potential sources of contamination such as rubbish, wastewater, and animals [27].

### 3.3. Sensory Appeal of Food

All the pupils in both Wa and Cape Coast schools had issues with food served to them. A majority (over 50%) of all the pupils in both schools complained of the meal having an offensive smell, partially cooked, presence of foreign materials, watery soup, and the color of the food not appealing. However, the response from the teachers in both schools disagreed with what the pupils reported except for the fact that they also agreed to the claim that the soup served to the pupils were light and watery (Table 7).

### 3.4. Taste Acceptability of Meals Served to Pupils of Wa and Cape Coast Schools

Findings in Table 8 revealed that although 27.8% of the pupils in Wa said their meals were tasty, and so they ate all their meals, 9.4% said, though tasty, they were unable to eat all the meal. Fifty per cent (50%) said the meals were not tasty but they ate all, while 12.8% said the meals were not tasty and they were unable to eat all though they were hungry. At Cape Coast 37.5% of the pupils claimed that their meals were tasty, and hence they ate all, while 4.5% said their meals were tasty but they were unable to eat. Thirteen point eight per cent (13.8%) claimed their meals were bad and so they could not eat the meal. 44.2% of the pupils in Wa ate all their meals though they were not tasty. A higher percentage of the pupils in Wa and Cape Coast ate all the meals though they were not tasty.

In one of the schools in Wa, almost all pupils in the upper primary rejected the food. This was because the food was not tasty according to the pupils. This was confirmed by the teachers including the head teacher, as they stated that the food served was less than what could be described as food for humans. Although the pupils rejected the food, the caterers consistently cooked the meals and this resulted in a lot of leftover foods. Where the surplus eventually ended up was unknown but it could best be guessed to end up in the homes of the cook or refuse dumps. In both Wa and Cape Coast municipal schools, none of the kitchen staff admitted that pupils and teachers ever complained about the meals they served to the pupils.

## 4. Conclusion

This study investigated the hygienic practices by school-going children and hygienic conditions in which food is prepared and served under the GSFP. The results indicated that the majority of the pupils do not practice good hand washing. The use of communal bowl for washing bowls and hands could be a possible route for faecal contaminants, especially where outdoor latrines did not have associated hand washing facilities. The conditions under which the meals were cooked, served, and eaten were generally fair, but for a couple of schools in Wa, the conditions were poor. This paper, therefore, recommends that the government through the assembly should provide potable drinking water to the schools. This can be done through the provision of tap water or boreholes to the schools so that kitchen staff in the schools can have access to potable water. The Municipal Assembly of Wa and Cape Coast should also ensure the supply of dustbins and Veronica Bucket to the schools especially those under the GSFP to improve food hygiene and sanitation in the schools.

## Figures and Tables

**Table 1:** Categorization of respondents.

Categories of respondents	Wa	Cape Coast	Total
Pupils	360	240	600
Head teachers/teachers	36	24	60
Kitchen staff	36	24	60
Total	432	288	720

**Table 2:** Sociodemographic features of respondents.

Category of respondents	Features	Descriptive features	Wa Municipal.	Cape Coast Metro.
Pupils	Age	Minimum	10	9
		Mode	13	12
		Mean	14	12
		Maximum	20	16
	Sex	Male	234	134
		Female	126	106

Kitchen staff	Educational level	Nonformal	21	—
		Basic	11	—
		Secondary or vocational	4	100
	Health certificate		15	16

**Table 3:** Responses given by participants regarding washing of bowls by the pupils.

Respondents	Wa		Cape Coast	
	Yes	No	Yes	No
Pupils	98.0	2.0	94.6	5.4
Teachers	93.0	7.0	92.2	7.8
Kitchen staff	33.3	66.7	13.0	87.0

**Table 4:** Hand washing techniques employed by the respondents in Wa and Cape Coast municipal schools.

Wa schools	Respondents					
	Pupils (*n* = 360)		Teachers (*n* = 36)		Kitchen staff (*n* = 36)	
	Yes (%)	No (%)	Yes (%)	No (%)	Yes (%)	No (%)
With soap under running water	7.78	92.22	27.78	72.22	11.11	88.89
Running water without soap	17.50	82.50	16.67	83.33	8.33	91.67
Bowl of water with soap	40.83	59.17	41.67	58.33	55.56	44.44
Bowl of water without soap	8.61	91.39	5.55	94.45	25.00	75.00
Do not wash hands	22.50	77.50	1.23	98.72	0.00	100.00
Others	2.78	91.22	1.10	98.90	0.00	100.00

Cape Coast schools	Pupils (*n* = 240)		Teachers (*n* = 24)		Kitchen staff (*n* = 24)	

With soap under running water	35.42	64.58	33.33	72.22	41.66	58.34
Running water without soap	25.83	74.17	16.67	83.33	20.83	91.67
Bowl of water with soap	16.66	83.34	29.16	70.84	25.00	75.00
Bowl of water without soap	10.42	89.58	16.67	83.33	12.50	87.50
Do not wash hands	11.67	88.33	4.17	95.83	0.00	100.00
Others	0.10	99.09	0.00	100.00	0.00	100.00

**Table 5:** Source of water for cooking meals in Wa and Cape Coast schools.

Water for cooking	Wa respondents			Cape Coast respondents		
	Pupils (*n* = 360) (%)	Teachers (*n* = 36) (%)	Kitchen staff (*n* = 36) (%)	Pupils (*n* = 240) (%)	Teachers (*n* = 24) (%)	Kitchen staff (*n* = 24) (%)
Pipe-borne water	100.0	100.0	91.7	100.0	100.0	100.0
Local well	2.5	1.0	15.3	0.5	1.0	15.0
Boreholes	78.2	82.3	41.7	70.10	80.3	40.7

**Table 6:** Hygienic conditions under which meals were prepared, served, and eaten in Wa Municipality and Cape Coast Metropolis schools.

	Response	Respondents					
		Pupils (%)		Teachers (%)		Kitchen staff (%)	
		Wa (*n* = 360)	Cape Coast (*n* = 240)	Wa (*n* = 36)	Cape Coast (*n* = 24)	Wa (*n* = 36)	Cape Coast (*n* = 24)
Prepared	Excellent	9.4	3.8	16.7	20.8	16.7	25.0
	Very good	16.7	10.8	16.7	16.7	11.1	25.0
	Good	34.7	37.0	33.3	20.8	22.2	41.7
	Fair	23.9	43.8	19.4	37.5	38.9	4.2
	Poor	15.8	4.6	13.8	4.2	11.1	4.2

	Excellent	0.0	0.0	2.8	4.1	2.8	4.1
	Very good	18.3	23.3	16.7	16.7	50.0	62.5
Served	Good	27.8	32.1	27.7	37.5	30.5	25.0
	Fair	30.8	40.8	22.2	29.1	11.1	4.1
	Poor	23.1	3.8	30.5	12.5	5.5	4.1

	Excellent	2.5	1.6	5.5	8.3	2.8	16.6
Eaten	Very good	8.6	5.8	25.0	20.8	22.2	33.3
	Good	17.2	40.4	52.8	50.0	30.5	25.0
	Fair	16.6	17.9	5.5	12.5	27.8	8.3
	Poor	55.0	32.5	11.1	8.3	19.4	16.6

**Table 7:** Complaints made by pupils and teachers about meals served by the kitchen staff.

Complaints about meal	Pupils (%)		Teachers (%)	
	Wa (*n* = 360)	Cape Coast (*n* = 240)	Wa (*n* = 36)	Cape Coast (*n* = 24)
Offensive smell	68.9	60.1	8.3	4.2
Partially cooked	81.4	82.5	19.4	12.5
Presence of foreign materials	79.4	71.4	13.9	4.2
Watery soap	79.7	85.2	66.7	92.0

**Table 8:** Acceptance of meal in Wa and Cape Coast municipal schools by the pupils.

Taste description of the meal	Percentage	
	Wa	Cape Coast
Tasty, hence I ate all my meal	27.8	37.5
Tasty, but unable to eat	9.4	4.5
Not tasty, but I ate all my meal	50.0	44.2
Not tasty, hence unable to eat	12.8	13.8
Total	100	100

## Data Availability

The data used to support the findings of this study are all included within the article. The questionnaires used to collect data can be made available by the corresponding author on reasonable request.
